# STRUGGLING FOR FOOD IN A TIME OF CRISIS: *A comment on Caplan (see pp 8–10 in this issue)*

**DOI:** 10.1111/1467-8322.12579

**Published:** 2020-06-04

**Authors:** Martin Caraher

**Affiliations:** ^1^ City, University of London

## Abstract

This commentary sets Caplan’s arguments about food banks and food poverty in the broader context of changes to the welfare state, the ‘charitization’ of state welfare and the need to address food poverty within a framework of dignity rather than charity.

 

## References

[anth12579-bib-0001] Barrie, J. 2019 People are sharing this open letter to MPs from a food bank volunteer about the struggles of people who rely on it. The i. https://inews.co.uk/news/politics/food-bank-volunteer-open-letter-mps-frank-field-heidi-allen/ (accessed 28 April 2020).

[anth12579-bib-0002] Buck, D. 2019 Public health spending: Where prevention rhetoric meets reality. The King's Fund. https://www.kingsfund.org.uk/.

[anth12579-bib-0003] Caplan, P. 2016 Big society or broken society? Food banks in the UK. Anthropology Today 32(1): 5–9.

[anth12579-bib-0004] Caraher, M. 2019 New food strategy for England. BMJ 366. 10.1136/bmj.l5711 31562123

[anth12579-bib-0005] Caraher, M. & R. Davison 2019 The normalization of food aid: What happened to feeding people well? Emerald Open Research 1(3). https://emeraldopenresearch.com/articles/1-3.

[anth12579-bib-0006] Caraher, M. & S. Furey 2018 The economics of emergency food aid provision. London: Springer.

[anth12579-bib-0007] De Schutter, O. 2013 The right to food in times of crisis. In Fair Just (ed.) Freedom from hunger: Realizing the right to food in the UK, 7–11. London: Just Fair.

[anth12579-bib-0008] Food Foundation 2020 COVID‐19: Latest impact on food. https://foodfoundation.org.uk/covid-19-latest-impact-on-food-2/.

[anth12579-bib-0009] Food Justice Campaign 2003 From food poverty to food justice: New bill gives ‘right to food’ for the first time. London: Food Justice Campaign.

[anth12579-bib-0010] Hammond, R.J. 1951 *Food*, vol. 1. *The growth of policy*. London: HMSO.

[anth12579-bib-0011] Sustain n.d. Achieving everyone's right to food. https://www.sustainweb.org/righttofood/.

[anth12579-bib-0012] Taylor‐Gooby, P. 2012 Beveridge overboard? How the UK government is using the crisis to permanently restructure the welfare state. Intereconomics 4: 224–229.

[anth12579-bib-0013] Thane, P. 2018 Divided kingdom: A history of Britain, 1900 to the present. Cambridge: Cambridge University Press.

